# Assessing the impact of silicon nanowires on bacterial transformation and viability of *Escherichia coli*†

**DOI:** 10.1039/d0tb02762f

**Published:** 2021-06-08

**Authors:** Michele Becce, Anna Klöckner, Stuart G. Higgins, Jelle Penders, Daniel Hachim, Caleb J. Bashor, Andrew M. Edwards, Molly M. Stevens

**Affiliations:** Department of Materials, Imperial College London London UK m.stevens@imperial.ac.uk; Department of Bioengineering, Imperial College London London UK; Institute of Biomedical Engineering, Imperial College London London UK; MRC Centre for Molecular Bacteriology and Infection, Imperial College London London UK; Department of Bioengineering, Rice University Houston Texas USA

## Abstract

We investigated the biomaterial interface between the bacteria *Escherichia coli* DH5α and silicon nanowire patterned surfaces. We optimised the engineering of silicon nanowire coated surfaces using metal-assisted chemical etching. Using a combination of focussed ion beam scanning electron microscopy, and cell viability and transformation assays, we found that with increasing interfacing force, cell viability decreases, as a result of increasing cell rupture. However, despite this aggressive interfacing regime, a proportion of the bacterial cell population remains viable. We found that the silicon nanowires neither resulted in complete loss of cell viability nor partial membrane disruption and corresponding DNA plasmid transformation. Critically, assay choice was observed to be important, as a reduction-based metabolic reagent was found to yield false-positive results on the silicon nanowire substrate. We discuss the implications of these results for the future design and assessment of bacteria–nanostructure interfacing experiments.

## Abstract 2

In this study nanowires were engineered and we investigated the interface between the biomaterial and bacteria, looking in detail at membrane rupture and cell viability.

## Graphical abstract

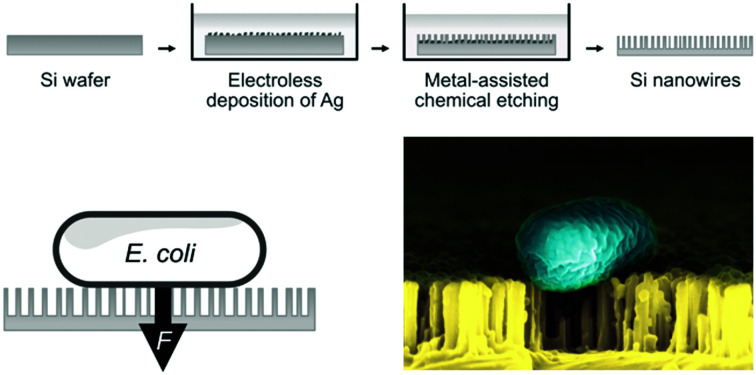

## Introduction

The concept of bacteria interfacing with high-aspect-ratio nanostructures has recently attracted interest because of possible applications in genetic manipulation, as well as generating antibacterial surfaces.

At a time of increasing antimicrobial resistance, materials and surfaces capable of repelling or killing pathogens through physical mechanisms have become a promising alternative to antibiotics, especially in the field of medical devices.^1,2^ Physical mechanisms avoid the use of chemical agents which become less effective with repeated exposure due to the development of antibacterial resistance.^3^ Antibacterial surfaces occur naturally, for example in the wings of cicadas, where the presence of sharp, vertically-aligned nanostructures can pierce bacterial cell envelopes leading to cell death.^4^ This has led to many biomimicry studies, which attempt to replicate similar nanostructures using engineered materials, both for medical and non-medical applications, such as marine anti-fouling coatings.^5^ Surfaces covered in vertically-aligned nanometre-scale protrusions, such as black silicon, have been shown to exhibit similar antibacterial properties to the cicada wing.^6–8^ The mechanisms driving this antimicrobial action remain an area of intense discussion in the literature.^9–13^ It can be challenging to isolate the explicit behaviour of surfaces such as silicon nanowires, as studies are intentionally convoluted with additional antibacterial agents such as metallic nanoparticles or polymers to maximise the killing effect.^14,15^ Studies which have examined the impact of silicon nanowires in isolation from surface chemistry suggest that the effect of nanowires alone is highly geometry-dependent, with some nanostructured surfaces exhibiting relatively low killing efficacy.^7^ Both scenarios are interesting; understanding how to engineer high-efficiency killing surfaces is important for antibacterial coating applications. Conversely, silicon nanowires that only partially disrupt the membrane can inform our understanding of when nanostructured surfaces are truly antibacterial, and are also useful in applications such as bacterial transformation. In the latter case, a nanostructured surface might be able to induce the direct uptake of genetic material into a cell.

Many similar discussions are taking place concurrently in the eukaryotic cell literature. Mammalian cells are frequently interfaced with surfaces covered with high-aspect-ratio nanostructures for a wide range of motivations (albeit not as a killing mechanism).^16^ As observed in bacterial cells,^17^ biological effects are strongly dependent on the precise size, shape and density of nanostructures.^16^ Recently, a number of studies have taken advantage of this geometry-dependent behaviour to target different cell types simultaneously, such as black silicon surfaces that kill clinically-relevant pathogens, while allowing the proliferation of eukaryotic cells.^18^ Nanostructured titanium was observed to inhibit pathogen growth while simultaneously promoting the osteogenesis of mesenchymal stem cells.^19^

We have previously reported how silicon nanoneedles, a type of vertically-aligned nanostructured surface, can be used to deliver nucleic acid into eukaryotic cells.^20^ Note: the process of introducing genetic material into a eukaryotic cell is called transfection, while for prokaryotic cells is termed transformation. Currently, the majority of bacterial transformation techniques are based on chemical transformation or electroporation.^21^ These approaches are essential to a range of basic research and biotechnology challenges, such as the production of insulin from genetically modified *Escherichia coli* or *Saccharomyces cerevisiae.*^22^ While freestanding nanostructures can improve transformation efficacies (when combined with electroporation or chemical poration),^21,23–25^ the long-term implications of the presence of high-aspect-ratio nanostructures remaining in the bacterial cell culture are unclear. In addition, even with these tools many bacterial strains remain difficult to transform.

Here, we report the engineering of surfaces of vertically-aligned silicon nanowires to act as a temporary interface for disrupting the bacterial envelope. The primary aim is to assess whether the nanostructure–eukaryotic cell interfacing approaches can be adapted for mechanically-based bacterial transformation. Analogous to our prior work with eukaryotic cells, we hypothesised that bacterial cells could undergo similar membrane disruption, and that by controlling the degree of mechanical interfacing we would be able to deliver plasmid DNA into the cytoplasm while still allowing recovery of the membrane. While ultimately we show that transformation is not viable with the current approach, our characterisation provides insight into the nature of the bacteria–nanowire interface, and highlights important considerations for future assay design, including the identification of confounding factors that might falsely over-estimate bacterial cell viability on silicon nanowire surfaces.

## Results and discussion

**Fig. 1:**
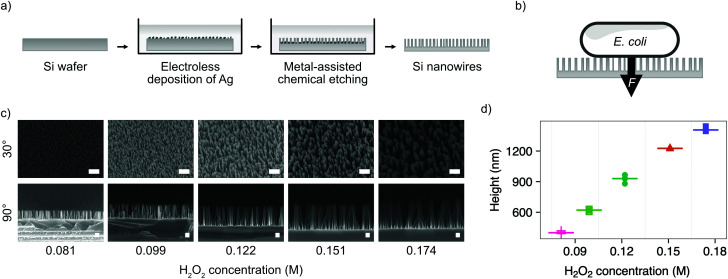
Fabrication of silicon nanowires for bacterial interfacing. (a) Illustration of silicon nanowire fabrication process. (b) Illustration of nanowire interfacing mechanism with *E. coli*, where the force *F* is either the result of cells settling under gravity or increased by centrifugation. (c) SEM micrographs showing the impact of varying the concentration of hydrogen peroxide (H_2_O_2_) in the etchant during MACE process. Samples imaged at 30° or 90°, as indicated, with no tilt correction applied. Scale bars are 1 μm for 30° and 200 nm for 90° images. (d) Impact of hydrogen peroxide concentration on resulting nanowire height. Horizontal lines represent the median of measurements. *n* = 5.

We chose the bacterium *E. coli* DH5α as a representative bacterial strain commonly used in molecular biology. *E. coli* cells have a rod-like phenotype, with typically sub-micrometre diameters. We optimised a microfabrication protocol for etching nanowires onto the surface of silicon wafers, to achieve the nanostructure dimensions and density required to interface cells on this length scale, illustrated in Fig. 1.

Silicon nanowires were etched from solid silicon wafers using metal-assisted chemical etching (MACE, described in the Experimental section).^26^ This process uses an incomplete layer of silver nanoparticles deposited onto the silicon surface using electroless deposition. These nanoparticles catalyse MACE reactions at the nanoparticle – silver interface, resulting in a rapid increase of the rate of anisotropic vertical etching into the surface of the wafer, leading to the formation of nanowires.^27^ The process was optimised by tuning the relative concentrations of hydrofluoric acid and hydrogen peroxide in the etchant solution (Fig. 1c). Increasing the concentration of hydrogen peroxide from 0.09–0.18 M results in the linear increase in median nanowire height from 399–1407 nm (Fig. 1d). Beyond concentrations of 0.18 M, the increased lateral etching rate results in over-etching of the surface and the collapse of adjacent nanowires into larger bundles (Fig. S1, ESI†). The stochastic nature of the silver nanoparticle deposition results in a distribution of nanowire diameters and spacing, with the median diameter initially increasing from 18–52 nm with increasing hydrogen peroxide concentration, before the increasing lateral etch results in a reduction in diameter beyond 0.12 M (Fig. S2, ESI†). The spacing between nanowires was in the range ∼81–162 nm. A concentration of 0.081 M was selected as providing the optimal balance between nanowire height (399 ± 10 nm) and uniformity (diameter 18 ± 4 nm, spacing 81 ± 35 nm), where the values in parenthesis refer to the median and median absolute deviation of the measurements.

**Fig. 2:**
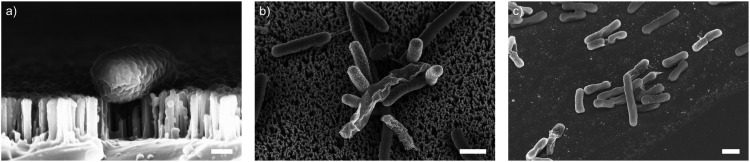
SEM micrographs of vertically-aligned silicon nanowires interfacing the bacteria *E. coli*. (a) Side-projection of single bacterial cell on nanowires, scale bar: 200 nm. (b) Top-down view of *E. coli* on nanowires (imaged at 30°, with no tilt correction), scale bar: 1 μm. (c) *E. coli* on flat silicon chip (imaged at 30°, with no tilt correction), scale bar: 1 μm.

As well as optimising the fabrication process to achieve reliable results, these nanowire dimensions are broadly consistent with the length scales of naturally-occurring high-aspect nanostructured surfaces, which vary in height from ∼100–250 nm, diameter ∼50–200 nm, and spacing 100–250 nm.^17^ Insight from the eukaryotic literature (albeit with differing dimensions, but similar relative scale) suggest that an individual nanostructure has a low probability of penetrating the cellular membrane under the influence of gravity alone,^16^ hence this length scale results in the bacteria being impinged by many tens of nanowires at the same time (Fig. 2).

Nanostructuring of the surface resulted in an increase in the water contact angle of the surface (from 81 ± 3° to 96 ± 3°, see Fig. S3, ESI†), indicating greater hydrophobicity of the nanostructured surface and within the range of other reported natural and artificially generated surfaces.^17^

Silver nanoparticle coated nanowires have been reported to have anti-bacterial properties.^14^ Hence, it is important to fully remove the catalyst in order to isolate the effect of the nanowires. The remaining silver nanoparticle catalyst was removed from the substrates by a further chemical etching step (see experimental methods). This removal was verified using X-ray photoelectron spectroscopy (XPS) (Fig. S4 and Table S1, ESI†).

The stability of the nanowires was tested by incubating substrates in different pH phosphate-buffered saline solutions for 0.5, 1, 2, 4 and 8 hours, after which no degradation was observed (Fig. S5, ESI†).

We investigated the efficacy of vertically-aligned nanowires as a bacterial transformation platform by attempting to deliver the plasmid pET-28a(+) (5369 bp) into *E. coli* DH5α cells. This commonly used plasmid carries a kanamycin resistant cassette which allows successfully transformed bacteria to be easily identified by growth on selective agar plates. Plasmids, suspended in water, were mixed into the bacterial cell suspension, before the combined mixture was dispensed onto both nanowire and flat silicon chips (dimensions 4 mm × 4 mm). Droplets of the cell suspension were carefully placed on top of the nanowire chips, to avoid wetting the well base or walls. Bacteria not confined to the top of the chip do not interface the nanowires, which could lead to the formation of subpopulations within the same culture, reducing the effective transformation efficacy observed.

We systematically investigated varying the interfacing force between the cells and nanowire chips. Spontaneous penetration of eukaryotic cell membranes by vertically-aligned nanostructures is rare,^16^ and often promoted by the application of external force *via* centrifugation.^20^ This force must be carefully optimised to avoid excessive cell death.^28,29^ Unlike eukaryotic cells, bacteria have both a cellular membrane and a peptidoglycan cell wall, the latter acting as an additional barrier to penetration. However, prior studies have shown that cell-wall containing microalgae (*Chlamydomonas reinhardtii*) can be successfully penetrated by microneedles using centrifugation.^30^ We therefore tested centrifuging cells onto chips at a range of different forces (0 × *g*, 1000 × *g*, 5000 × *g* and 12 500 × *g*, corresponding to a force acting on a single cell body of roughly 0 pN, 9 pN, 47 pN, 117 pN, respectively). Cells were then incubated for 1 h in SOC media to promote recovery and allow cells to express kanamycin resistance, before being plated on selective agar plates with and without kanamycin. Despite the increased mechanical force applied to the bacterial cells, none of the tested conditions resulted in the uptake of the plasmid, observed as a lack of colony formation on the antibiotic-containing plates (Fig. S6, ESI†). As multiple controls, bacteria were also incubated on flat and nanowire chips without centrifugation, again no transformation was observed. Similarly, the capability of the bacterial strain to propagate the plasmid was confirmed using chemically-competent *E. coli* DH5α cells and a standard heat-shock transformation protocol (Fig. S7, ESI†). This bacterial strain, as well as the plasmid, are commonly used and suitable for transformation, as confirmed by the numerous colonies we observed on the selective plates containing kanamycin (Fig. S7, ESI†). The bacterial growth on non-antibiotic containing agar plates confirmed that a proportion of bacterial cells survive contact with the silicon nanowires, even under large interfacing forces.

**Fig. 3:**
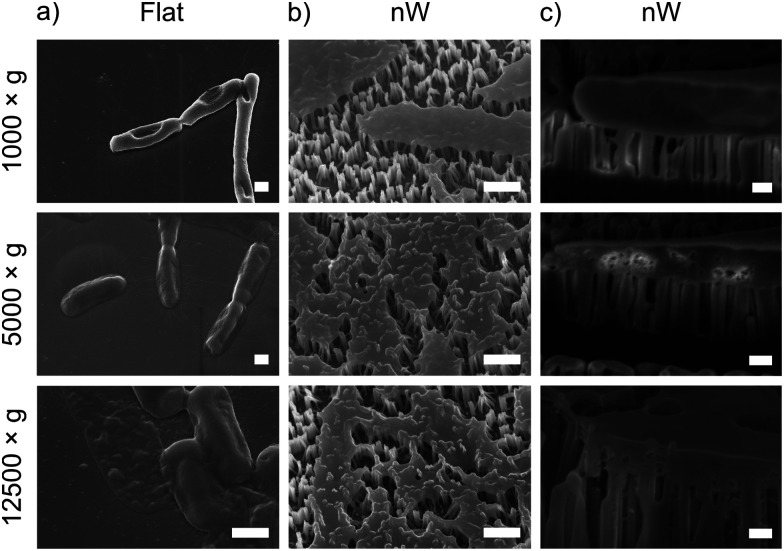
Representative SEM and FIB-SEM micrographs of *E. coli* seeded on nanowire and flat chips, imaged after centrifugation. (a) and (b) SEM micrographs (imaged at 30°, with no tilt correction), scale bars: 500 nm. (c) FIB-SEM micrographs (imaged at 90°), scale bars: 200 nm.

To understand these results, we considered whether cells are forced from the nanowire chips during centrifugation, which would prevent interaction with the surface. We used scanning electron microscopy (SEM) to confirm that bacterial cells remain on the nanowire chips post centrifugation (Fig. 3). SEM images reveal that bacterial cells remain on the chips and suggest that centrifugation leads to severe membrane damage. Notably, this deformation was observed for cells on both nanowire and flat chips (Fig. 3), and not in non-centrifuged samples. We used an osmium-based cell fixation protocol (see Methods), combined with focused ion-beam scanning electron microscopy (FIB-SEM) to image cross-sections through the bacteria–nanowire interface. Cells were fixed, dehydrated and resin-embedded using an adapted version of our previously reported protocol for eukaryotic cells,^31^ which aims to minimise dehydration artefacts that can distort cellular shapes.^32^ Despite this, care must still be taken while interpreting single-cell images, however, these micrographs allude to a general flattening of bacterial cells with increasing centrifugation speed. This provides evidence of increasing membrane disruption with interfacing force. In some cases, the bacterial cell envelope can be seen to wrap around the nanowire surface (Fig. 3c). The degree of membrane engulfment appears to increase with increasing interfacing force.

**Fig. 4:**
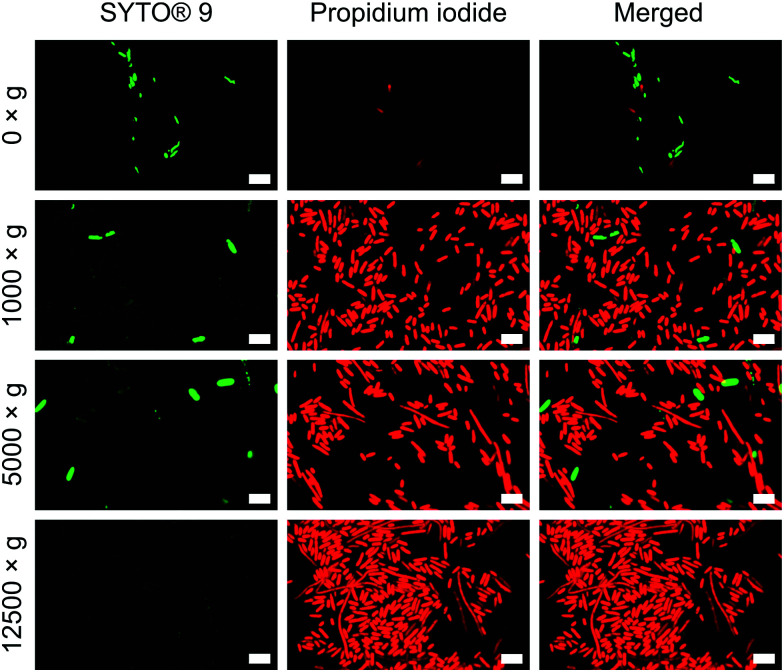
Propidium iodide uptake confirms impaired bacterial membranes of *E. coli*. Bacteria were incubated with both dyes immediately after centrifugation. *n* = 3. Scale bars: 5 μm.

To verify the impact of both nanowire interfacing and centrifugation, we used a bacterial viability kit (Live/Dead® BacLight™) to further characterise membrane integrity. The assay comprises two fluorescent dyes: SYTO® 9, which can penetrate intact cell membranes; and the membrane impermeant propidium iodide (PI).^33^ Cells with intact cell membrane appear green, those with disrupted membranes appear red (Fig. S8, ESI†). This assay confirmed that the centrifugation of cells onto nanowires resulted in significant permeabilization of the bacterial cell membranes (Fig. 4), but to a far lesser extent for non-centrifuged chips. Similar membrane disruption is observed for cells centrifuged onto flat chips (Fig. S9, ESI†), suggesting that the mechanical force of the centrifugation, rather than an intrinsic effect of the silicon nanowires, is the dominant promoter of membrane disruption. Note: the lower apparent cell density on non-centrifuged chips is due to a lack of interfacing force to keep bacteria on the surface of the chip.

**Fig. 5:**
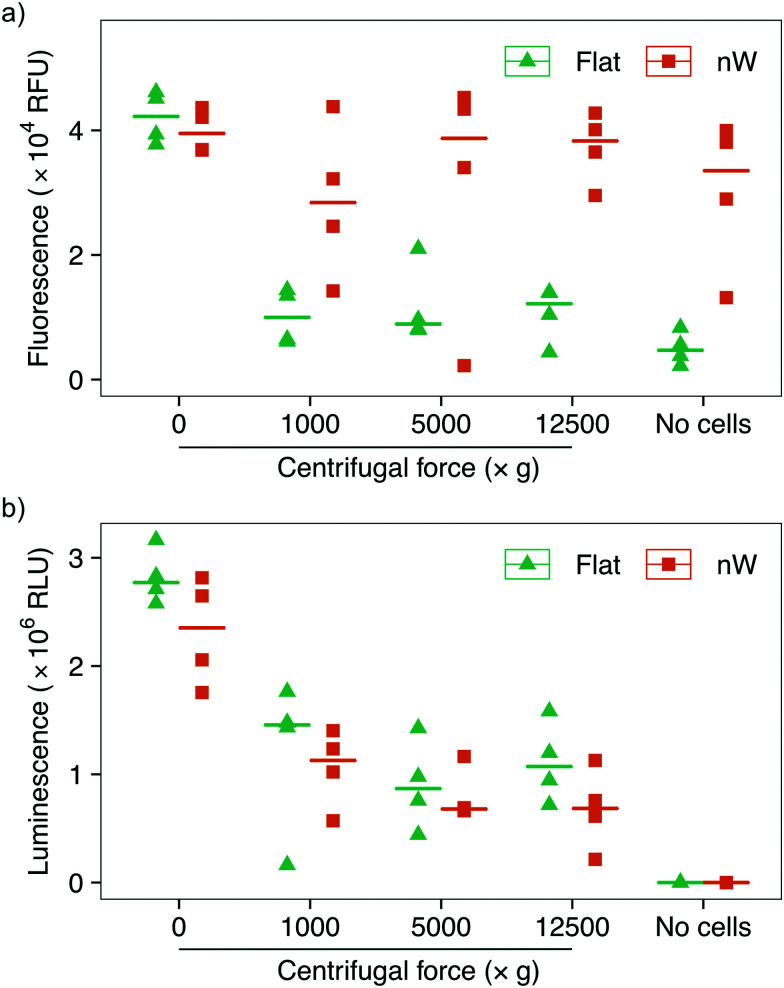
Cell viability assays of *E. coli* after centrifugation on silicon nanowire and flat chips. (a) alamarBlue™ assay data: the nanowire chip alone acts to reduce the reagent, making the assay unsuitable for the evaluation of bacterial survival. (b) ATP-detecting BacTiter-Glo™ assay shows reduction in cell viability with increasing centrifugal force. Horizontal lines indicate the median value. *n* = 4.

Assays based on membrane permeable and impermeable dyes are frequently used in the interfacing literature to determine the proportion of live and dead cells,^3^ however membrane disruption alone is not an exclusive marker of cell death.^34,35^ We wanted to determine whether the increased uptake of the membrane-impermeant stain was the result of temporary pore formation from which the cell would recover, or due to severe cell damage that would ultimately result in cell death. We first attempted to use a metabolic-based assay (alamarBlue™), which quantifies cell viability by detecting the chemically-reducing environment present inside living cells. Cells were incubated with the membrane-permeable dye resazurin, which is irreversibly reduced to resorufin in the cell cytoplasm, forming a highly fluorescent compound. The relative change in fluorescence between the starting and reduced states is used as an indication of cell viability. This assay showed a reduction in cell viability for centrifuged cells on flat substrates compared to no centrifugation (Fig. 5a), consistent with the results above. Nevertheless, cells on silicon nanowires showed no change in fluorescence, superficially suggesting that nanowires do not reduce cell viability. However, an additional control demonstrated that the silicon nanowire chip appears to promote the reduction of the dye, even in the absence of bacteria. This effect has previously been observed during eukaryotic cell culture on porous silicon substrates.^36^ Ultimately, this makes it impossible to evaluate this assay's results in the context of silicon nanowires and excludes its use to determine changes in cell viability.

To overcome this limitation, we tested another metabolic assay (BacTiter-Glo™). This assay is based on the quantification of adenosine triphosphate (ATP), an indicator of metabolically active cells. A luciferase enzyme catalyses the reaction of luciferin in the presence of ATP, molecular oxygen and the assay buffer, resulting in light emission. The luminescent signal is directly proportional to the concentration of ATP and hence the number of viable cells. Control experiments in the absence of bacteria confirmed that this reaction is not catalysed by the silicon nanowires, allowing the effect of nanowires on bacteria to be isolated (Fig. 5b). These results show a systematic reduction in cell viability between non-centrifuged and centrifuged cells on both nanowire and flat chips. This assay also suggests a subtle systematic reduction in the viability of cells cultured on nanowires *versus* flat substrates.

Since *E. coli* is a Gram-negative bacterium, to further understand the observed changes in viability we tested *Staphylococcus aureus*, a common pathogenic Gram-positive bacterium, using the same silicon nanowire substrates and centrifugation protocol. Similar to *E. coli*, a systematic decrease in cell viability was observed with increasing centrifugation speed, and again a subtle reduction in viability between flat and nanowire substrates was observed (Fig. S10, ESI†).

## Experimental

### Fabrication of silicon nanowires

Boron-doped p-type silicon wafers, diameter 100 mm, resistivity 0.01–0.02 Ω cm and 〈100〉 ± 0.5° orientation (University Wafers, USA), were used as the base substrate. Wafers were oxygen-plasma cleaned (300 W, 13.56 MHz, 10 min, Diener Plasma cleaner, Germany) before undergoing metal-assisted chemical etching (MACE).^26^ De-ionised (DI) water was used for all aqueous fabrication processes, unless specified otherwise. Any native silicon oxide layer present on the surface of a wafer was removed using a 2.83 M solution of hydrofluoric acid (HF) (semiconductor grade, Aldrich/Fisher, USA), immersing the wafer for 1 min. Immediately after native oxide stripping, each wafer was immersed into an electroless deposition (ELD) solution, comprising 2.83 M HF and 0.02 M AgNO_3_ (Sigma-Aldrich Fluka, USA, used as received). Wafers were immersed for 1 min, under gentle agitation, in order to homogenise the silver nanoparticle deposition across the wafer surface. After silver deposition, the wafer was immersed in ∼800 mL of water, to stop the ELD process, before successive rinsing with a gentle flow of water, then isopropyl alcohol (IPA, Fisher Scientific, USA), and finally blow-dried with a stream of N_2_ gas. After visual inspection, each wafer was immersed into its respective MACE solution, comprising HF and hydrogen peroxide, H_2_O_2_ (Sigma-Aldrich, USA). For optimisation, experiments the concentration of HF acid was fixed at 2.83 M, and the concentration of H_2_O_2_ varied between 0.081 M and 0.802 M. The optimised process conditions used in subsequent biological assays used an H_2_O_2_ concentration of 0.081 M. Wafers were immersed in the etchant solution for 2 min, followed by immersion in ∼800 mL of water to halt the process, rinsing with a gentle flow of water, then with IPA, and drying under ambient conditions in a fume hood. Residual silver nanoparticles on the surface of the substrate were removed by immersing each wafer in ∼100 mL of gold etchant (Aldrich, USA) for 10 min, followed again by the washing and drying steps described above. After fabrication, each silicon wafer was diced into 4 mm-side square chips (DISCO, Japan). Before use in biological assays, each chip was treated with oxygen plasma (100 W, 40 kHz, 20 min, GaLa Instrumente, Germany), then with piranha solution (3 : 1 v/v H_2_SO_4_ : H_2_O_2_, 1 h, reagents from Sigma-Aldrich, USA). Each chip was then rinsed with water and blow-dried with N_2_ gas. Water contact angle measurements were performed using a goniometer (Krüss FM40 Easydrop, Germany), using 1 μL of deionised water. Contact angles were fitted using the provided software (Drop Shape Analysis (DSA) for Windows, version 1.90.1.14, Krüss, Germany), with further analysis and plotting performed using R (R Foundation for Statistical Computing, Vienna, Austria).

### X-Ray photoelectron spectroscopy of silicon nanowires

Elemental composition and absence of silver was assessed through X-ray photoelectron spectroscopy (XPS) on a ThermoFisher K Alpha^+^ XPS system (Waltham, MA, USA). To acquire the overall elemental composition, a survey of 4 scans was performed with a constant analyzer energy of 200 eV, dwell time of 25 milliseconds, 0.5 eV step size and an X-ray spot size of 400 μm. For detailed elemental information for silver, spectra of 10 scans were obtained using constant analyzer energy of 20 eV, dwell time of 50 milliseconds, 0.1 eV step size and an X-ray spot size of 400 μm. Carbon peak was used as reference, with a value of 284 eV for adventitious carbon. Spectra data was analyzed using Avantage software V5.9925, (Thermo Scientific, Waltham, MA, USA).

### Stability test of silicon nanowires

Each nanowire chip was put into the well of a 96-well plate (Corning, USA). Then, 200 μL per well of Dulbecco's phosphate-buffered saline (DPBS, Gibco, USA) were added, used either as purchased, or pH-adjusted with 2 M NaOH and 5 M HCl aqueous solutions. At each time point, the liquid was removed, the chip taken out of the well, and blow-dried with a gentle stream of dry N_2_ gas. The chips were then mounted onto scanning electron microscopy (SEM) stubs (Electron Microscopy Sciences, USA) with carbon tape (Agar Scientific, UK) and imaged in an electron microscope (Zeiss, Germany), without any coating. The height, diameter and spacing of patterned nanowires were measured and analysed using the software packages FIJI and R respectively (R Foundation for Statistical Computing, Vienna, Austria), with the package “tidyverse”.^37,38^

### Cultivation and preparation of bacteria

The cultivation and preparation of bacteria for all experiments is done as described in the following section. A volume of 5 mL of lysogeny broth (LB), also referred to as luria broth (Fisher Scientific, USA) was inoculated with *Escherichia coli* DH5α (NEB, USA) and grown overnight at 37 °C, shaking (170 rpm). Fresh LB media (5 mL) was inoculated with 1% v/v of the overnight culture and incubated at 37 °C shaking until OD600 of 0.25. A volume of 1 mL of the culture was harvested at 16 200 × *g* for 3 min and the pellet was resuspended in 10 μL water. Experiments performed with *Staphylococcus aureus* JE2 were executed in the same way but bacteria were cultivated in tryptic soy broth (TSB; BD Biosciences, USA).

### SEM and FIB-SEM imaging of bacteria on nanowires

For both SEM and FIB-SEM samples, cells were first prepared as described above and 3.5 μL of the culture was spotted onto either nanowires or flat silicon chips and centrifuged at 1000 × *g*, 5000 × *g* or 12 500 × *g*. In the case of SEM samples, cell fixation was performed in glutaraldehyde (2.5% v/v in 0.01 M PBS) for 30 min at room temperature, followed by sample washing three times in PBS (0.01 M). Samples were then dehydrated in ethanol aqueous solutions (10% v/v, 30% v/v, 50% v/v, 70% v/v, 90% v/v, 100% v/v), for 5 min for each concentration. The samples were then attached to aluminium stubs (Electron Microscopy Sciences, USA) with double-sided carbon tape (Agar Scientific, UK). They were then coated with a 15 nm-thick layer of chromium (Quorum Technologies, UK) and imaged with a LEO Gemini 1525 field emission gun scanning electron microscope (FEG SEM; Carl Zeiss Microscopy, Germany). Unless otherwise specified, micrographs were taken at a 30° tilt angle.

In the case of FIB-SEM, bacteria were centrifuged on the chips for either 1000 × *g* for 5 min, 5000 × *g* for 5 min, or 12 500 × *g* for 20 min. Samples were then placed into a 96-well plate, immersed in 0.1 mL of a 2.5% v/v solution of glutaraldehyde in PBS and incubated at room temperature for 30 min. After this, they were washed three times in PBS and left in the fridge at 4 °C overnight. Then, PBS was removed, and the samples washed twice, for 5 min each time, in a 0.1 M cacodylate aqueous solution. For better handling, chips were successively transferred into a 24 well plate. They were then fixed in 2.5% v/v glutaraldehyde in 0.1 M cacodylate for 1 h, and washed twice in water, for 5 min each time. They were stained in a 1% v/v aqueous solution of OsO_4_ for 1 h and washed twice in water, for 5 min each time. After this, samples were incubated in a 1% v/v aqueous solution of tannic acid (previously filtered with a 0.2 μm syringe filter) for 1 h and washed twice in water, for 5 min each time. Samples were then stained in a 1% v/v aqueous solution of uranyl acetate (previously filtered with a 0.2 μm syringe filter) for 2.5 h and washed twice in water, for 5 min each time. Afterwards, samples were dehydrated, by incubating twice for 5 min each in 20% v/v, 30% v/v, 50% v/v and 70% v/v ethanol aqueous solutions, and left in the 70% v/v solution overnight. After this, the dehydration was completed with the same incubation procedure in 80% v/v, 90%, v/v and 100% v/v ethanol aqueous solutions. The last step in 100% ethanol was repeated another two times. Samples were then embedded in resin (Epoxy Embedding Medium kit, Sigma-Aldrich, USA), 2.5 h in each of 3 : 1, 2 : 1, 1 : 1 ethanol : resin v/v solutions, then overnight in a 1 : 2 solution. Finally, the sample was put in pure resin for 2.5 h, twice. The resin was then cured at 60 °C for 48 to 72 h. The samples were then attached to aluminium stubs (Electron Microscopy Sciences, USA) with double-side carbon tape (Agar Scientific, UK), coated with a 15 nm-thick-layer of chromium (Quorum Technologies, UK) and imaged with a Zeiss Auriga Cross Beam FIB-SEM (Carl Zeiss Microscopy GmbH, Germany). Micrographs were taken at a 54° tilt angle, with no tilt correction applied.

### Transformation assay

For transformation assays, cells were prepared as described in the section above (“Cultivation and preparation of bacteria”) and 1000 ng plasmid DNA (pET28b, Novagen, Merck, Germany) were added. A volume of this mixture (3.5 μL) was added to the chips and centrifuged at 1000 × *g*, 5000 × *g*, or 12 500 × *g* for 5 min. Immediately after, 150 μL SOC media was added and incubated for 1 h at 37 °C, shaking. After the incubation 50 μL each was transferred on selective (25 μg mL^−1^ kanamycin) or non-selective LB agar plates and incubated overnight at 37 °C. As a positive control, chemically competent *E. coli* DH5α prepared as described before were used.^39^ Cells were incubated with 1000 ng pET28b plasmid DNA on ice for 30 min and heat shocked for 60 s at 42 °C. After a 2 min incubation time on ice 950 μL SOC media was added and incubated for 1 h at 37 °C, shaking. As performed for the nanowires, 50 μL each of the culture was plated on selective and non-selective LB agar plates. Plates were incubated at 37 °C overnight and colony forming units were counted.

### Bacterial cell viability assays

#### Live/Dead®

Bacteria were treated as described in the section above (“Cultivation and preparation of bacteria”). After centrifugation, chips were transferred into 96 well plates and incubated with the Live/Dead® BacLight™ kit (L7012, Thermo Fisher, USA), as instructed by the manufacturer. Equal volumes of SYTO® 9 and PI were combined, added to the samples and incubated for 15 min at room temperature. Afterwards, chips were taped to a microscope slide and covered with a cover slip. Images were taken using a fluorescence microscope using Axio Imager. A2 (Carl Zeiss Microscopy GmbH, Germany).

#### alamarBlue™

Bacteria were treated as described in the section above (“Cultivation and preparation of bacteria”). After centrifugation, chips were transferred to a 96 well plate and 100 μL alamarBlue™ (DAL1025, Thermo Fisher, USA) solution (diluted 1 : 10 in PBS) was added. Samples were incubated overnight at 37 °C. After the incubation time, the solution was transferred into a black 96 (Greiner Bio-one, Austria) well plate and fluorescence signal was measured using a Tecan infinite 200 pro microplate reader. This experiment was performed twice, each with two technical replicates under identical conditions (*n* = 4).

#### BacTiter-Glo™

Bacteria were treated as described in the section above (“Cultivation and preparation of bacteria”). Chips were incubated with the BacTiter-Glo™ (Promega, UK) mixture in 96 well plates, as recommended by the manufacturer. After the incubation time, the solution was transferred into a white 96 well plate (Costar, UK) and luminescence was measured using a Tecan infinite 200 pro microplate reader. Luminescence values were normalised to the background intensity measured in adjacent empty wells. Data was analysed and plotted using the software package R. This experiment was performed twice, each with two technical replicates under identical conditions (*n* = 4).

## Conclusions

Considering the results from these multiple assays together, we can begin to elucidate the impact of silicon nanowires and interfacing force on *E. coli*. We find that simply seeding *E. coli* onto silicon nanowires results in little change in the permeability of the cell membrane, and no observable cell deformation (*via* SEM imaging), compared to flat silicon chips. An apparent small systematic reduction in cell viability may be present when comparing nanowire to flat chips under all conditions (Fig. 5b), including no additional/external interfacing force, with a similar pattern of behaviour observed with *S. aureus* (Fig. S10, ESI†). However, given the subtlety of the shifts observed, further experiments are required to verify this. A slight reduction would be consistent with previous observations with *E. coli*, where cells were interfaced with silicon nanowires of broadly comparable dimensions, which show that under some circumstances nanowires alone only exhibit minor antimicrobial behaviour.^7,40^

It is clear that centrifugation of *E. coli* bacterial cells onto silicon nanowire chips results in severe membrane disruption, as indicated by the intake of membrane impermeable dye and cell collapse observed *via* SEM. However, control experiments with flat chips suggest the majority of this effect can be attributed predominantly to the external applied force, rather than the inherent geometry of the substrate. Interestingly, even at a 12 500 × *g*, which corresponds roughly to interfacing forces in excess of 117 pN per cell (and resulting in very large localised pressure at the nanowire tips), nanowires do not result in complete eradication of the bacteria, with residual metabolic activity still observed for both nanowire and flat chips. Similarly, although viability is again reduced with centrifugation speed, a viable population of *S. aureus* remain on silicon nanowires at 12 500 × *g* (Fig. S10, ESI†), suggesting nanowires and interfacing force alone are not sufficient to completely irradicate an entire population. This observation is consistent with a recent report from Jenkins *et al.*,^11^ which studied both *E. coli* and *S. aureus* on titanium dioxide nanopillars, where they attributed reductions in cell viabilities to nanopillar-induced oxidative stress, rather than mechanical lysis. Interestingly, they observed a greater degree of penetration in electron microscopy imaging without the need for centrifugation, indicating that nanostructure geometry and material composition remain important factors. One potential limitation of our approach is that, while care was taken to minimise cells forming sub-populations in the chip-well plate configuration, it is possible that some cells were able to avoid direct contact with the chip surfaces, resulting in cell survival. In addition, while we observed most *E. coli* tended to lie flat on the chip surfaces, some cells were observed stacked on top of others (as seen in Fig. 2b and c), which may offer protection from the interfacing force. Another potential area for further investigation is the effect of nanowire geometry. In the eukaryotic literature, subtle differences in nanostructure geometry can strongly impact cellular response.^41^ In this work, we have attempted to replicate the dimensions of naturally occurring surfaces, however exploring parameters such as nanowire spacing may offer access to a greater range of bacterial cell responses.

The lack of observed delivery of pET28b plasmid, in spite of both clear membrane disruption and incomplete killing on centrifuged chips, suggests either incomplete plasmid delivery (where the plasmid fails to pass through both cell membrane and wall); or, alternatively, that the induced pores are large enough for propidium iodide to pass through, but not the plasmid. Further studies could consider membrane disruption as a function of nanowire geometry, or work to further model the bacterial cell–nanowire interface as a function of interfacing force.

We found that reliance upon a single assay or imaging-modality alone may lead to misleading conclusions. Considering our cell viability staining or SEM imaging results in isolation might lead to the conclusion that centrifuging cells onto silicon nanowires results in a highly effective killing surface, however the results from the metabolic assays suggest a significant subpopulation remains. The reduction of a fluorescent marker in the presence of silicon nanowires alone resulted in a potentially misleading experimental artefact. Furthermore, the lack of standardised interfacing protocols across the field may be reflected in the relative inconsistency in reported killing efficiencies in the literature. Our findings do not preclude the antimicrobial activity of silicon nanowires, but they do highlight that any such effects are highly parameter and measurement dependent, a potential issue for the real-world adoption of nanostructure-based surfaces. We suggest that future studies take into account the limitations of existing assays and adopt a combination of material and biological characterisation techniques, to further explore the bacteria–nanowire interface.

## Conflicts of interest

There are no conflicts to declare.

## Supplementary Material

TB-009-D0TB02762F-s001
