# Supplementary material for: Assessing the impact of silicon nanowires on bacterial transformation and viability of Escherichia coli
Source: J Mater Chem B. 2021 Jun 8;9(24):4906–14. doi: 10.1039/d0tb02762f (PMC8221286; doi:10.1039/d0tb02762f)
Supplement: TB-009-D0TB02762F-s001 [file TB-009-D0TB02762F-s001.pdf]

Electronic Supplementary Material (ESI) for Journal of Materials Chemistry B.
This journal is © The Royal Society of Chemistry 2021

Supplementary Information

Assessing the impact of silicon nanowires on bacterial transformation and viability of *Escherichia coli*

Michele Becce[a,b,c‡], Anna Klöckner[a,b,c,d‡], Stuart G. Higgins[a,b,c‡], Jelle Penders[a,b,c], Daniel Hachim[a,b,c], Caleb J. Bashor[e], Andrew M. Edwards[d], Molly M. Stevens[*a,b,c]

[a] Department of Materials, Imperial College London, London, United Kingdom

[b] Department of Bioengineering, Imperial College London, London, United Kingdom

[c] Institute of Biomedical Engineering, Imperial College London, London, United Kingdom

[d] MRC Centre for Molecular Bacteriology and Infection, Imperial College London, London, United Kingdom

[e] Department of Bioengineering, Rice University, Houston, Texas, United States of America

* Corresponding author

Email: m.stevens@imperial.ac.uk

‡ These authors contributed equally to the manuscript

Supplementary Figures

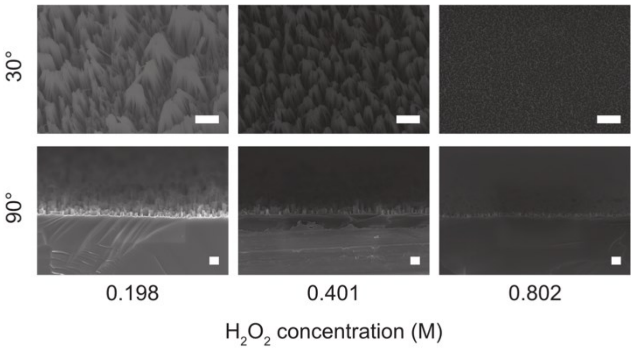

Fig S1. Over-etching of nanowires occurs for high hydrogen peroxide concentrations. SEM micrographs with top row, imaged at 30°, with no tilt correction, scale bars: 1 µm; and bottom row imaged at 90°, scale bars: 200 nm.

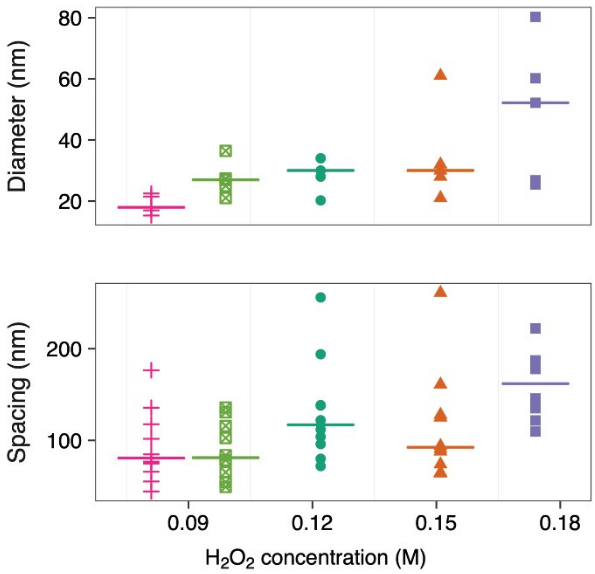

Fig S2. Impact of hydrogen peroxide concentration in MACE etchant on nanowire diameter and spacing. Measurements made from SEM images, horizontal lines represent the median of measurements. n = 5 and n = 10 for diameter and spacing measurements respectively.

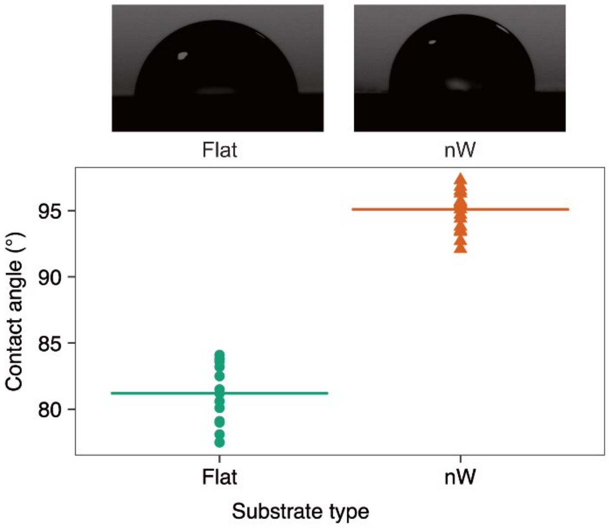

Figure S3. **Contact angle measurement of flat silicon and silicon nanowire substrates, as selected for bacterial assays.** Nanostructuring of the surface results in an increase in the water contact angle from 81 ± 3 ° to 96 ± 3°, (reported as median with median absolute deviation). n = 15, with 3 droplets on different regions of same wafer, fitted 5 times each.

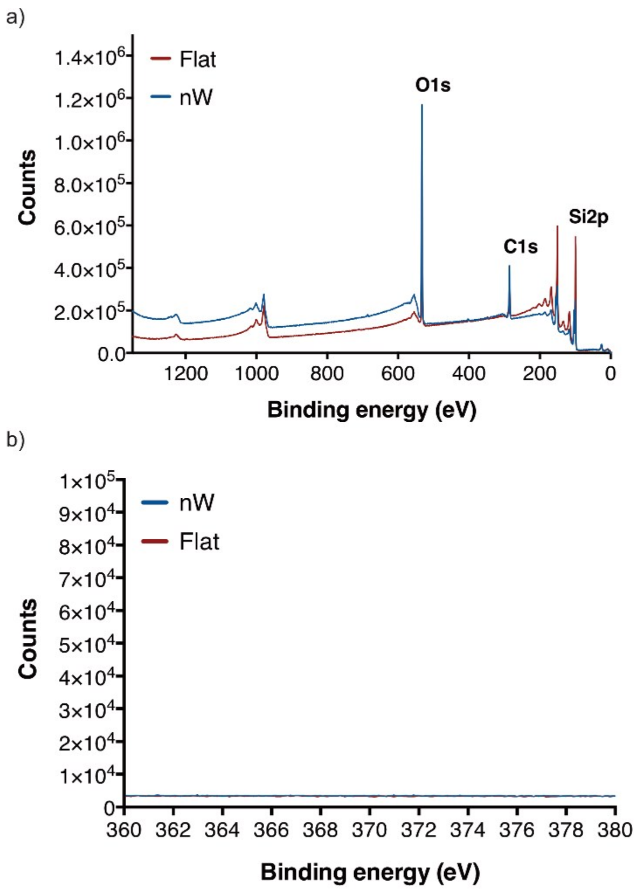

Figure S4. XPS data of flat silicon nanowire substrates. a) XPS survey showing elemental composition from flat silicon substrates (red) and silicon nanowire substrates (blue). b) Silver XPS spectrum from flat silicon substrates (red) and silicon nanowire substrates (blue).

	**Atomic %**		
	**Si2p**	**O1s**	**C1s**
**Flat**	53.01	31.82	15.17
**nW**	33.34	38.43	28.22

Table S1. XPS elemental composition (atomic %) from flat silicon substrates and silicon nanowire substrates.

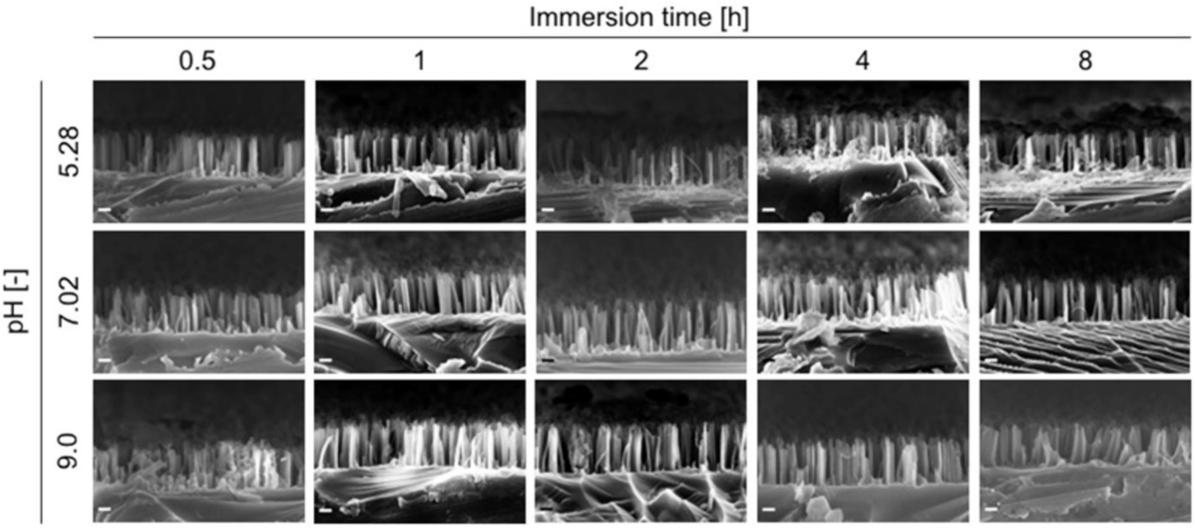

Fig S5. Multi-panel plot showing stability of nanowires in different pH and time. SEM micrographs of the samples used for the degradation studies. Sample view tilted 90°. Scale bars: 100 nm.

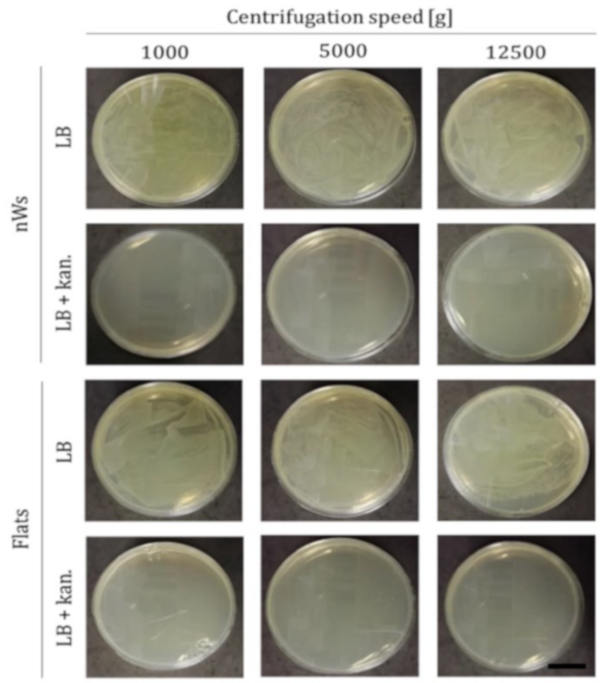

Fig S6. Assay to determine the efficacy of nanowire-mediated bacterial transformation. *E. coli* DH5α were incubated on selective and non-selective agar plates after centrifugation in the presence of plasmid DNA. The absence of colony forming units on kanamycin containing plates confirms that bacteria did not uptake the plasmid, whereas bacterial growth on non-selective plates can be detected. n = 3, scale bar: 25 mm.

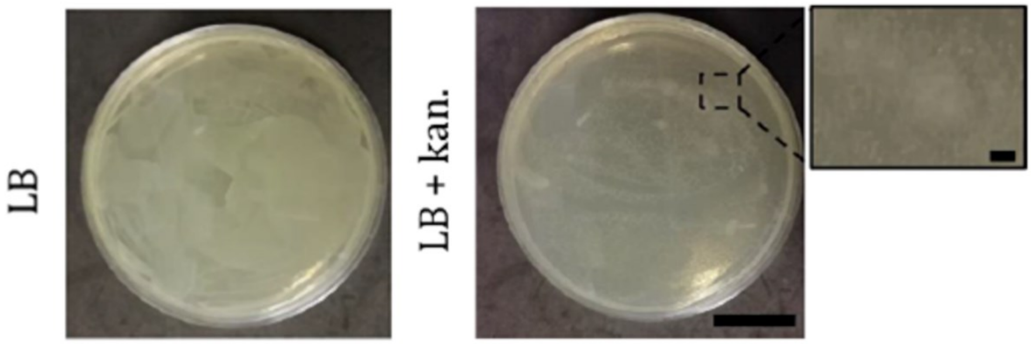

Fig S7. Control experiment to confirm the chemical transformation of *E. coli* DH5α. *E. coli* colonies are detectable on both selective and non-selective agar plates and served as a control. n = 1. Scale bar: 25 mm, inset: 2.5 mm.

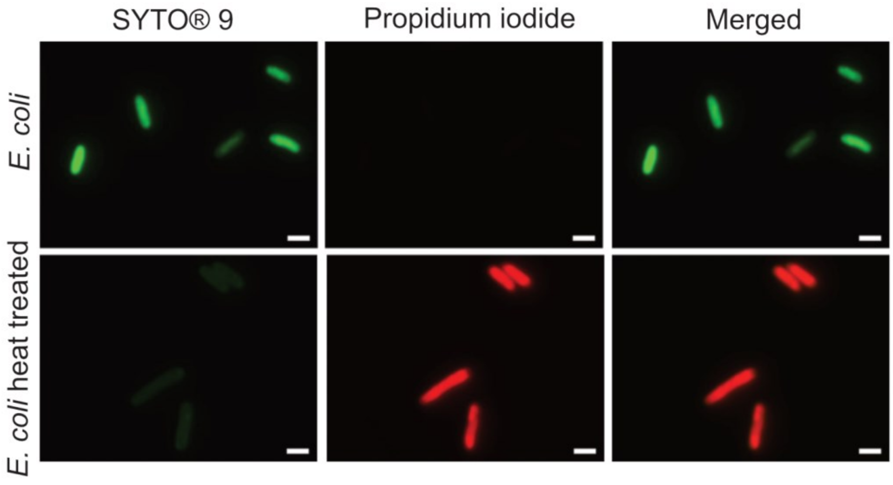

Fig S8. Fluorescence microscopy of *E. coli* untreated or heat inactivated. Scale bars: 2 µm. n = 1.

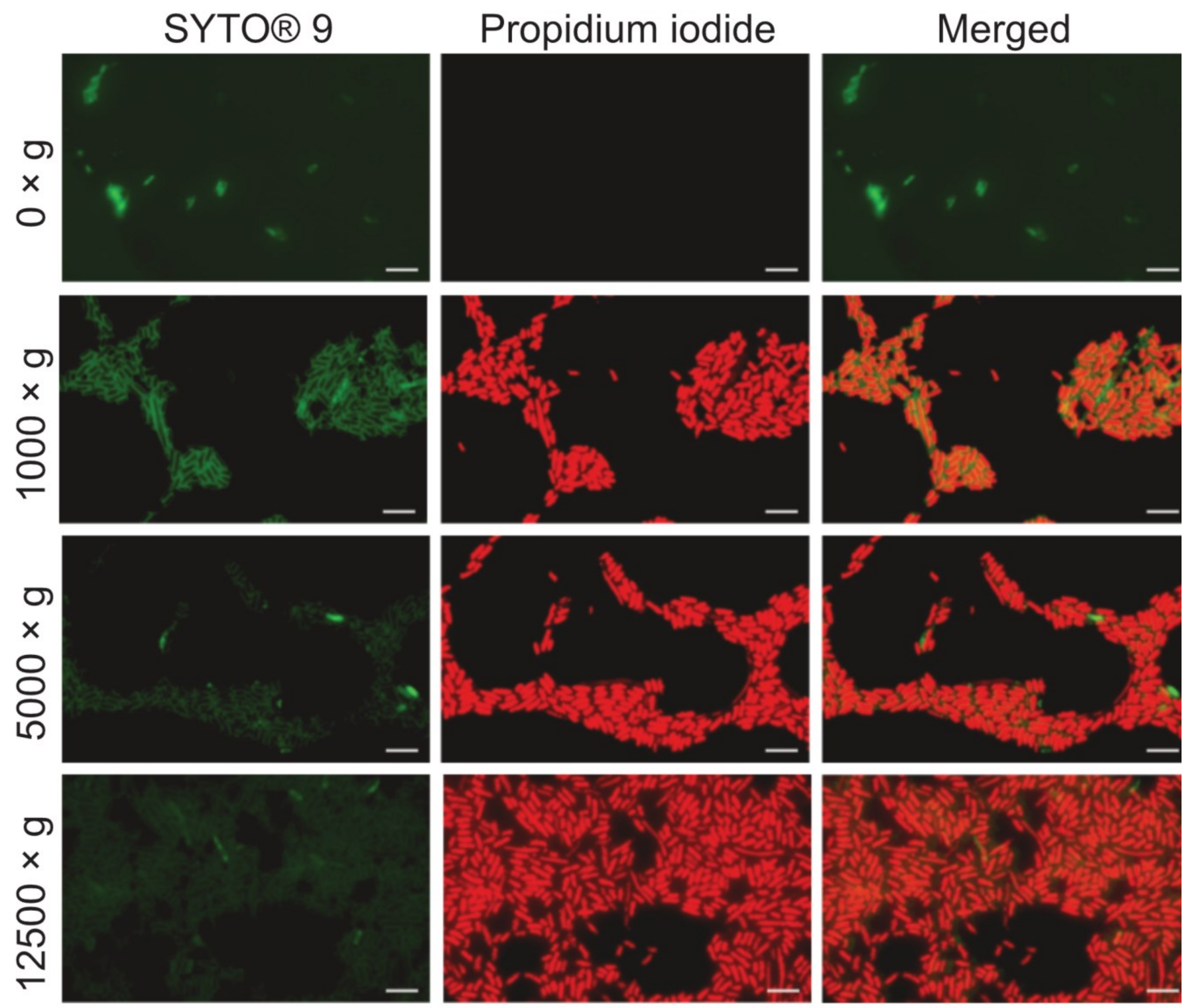

Fig S9. Fluorescence microscopy of *E. coli* seeded on a flat substrate with or without centrifugation steps. At 1000 × g, 5000 × g, and 12500 × g, the SYTO® 9 dye is capable of entering damaged cells, hence the low-level emission. Note: the lower apparent cell density on non-centrifuged chips is due to a lack of interfacing force to keep them on the surface of the chip. Scale bars: 5 µm. n = 3.

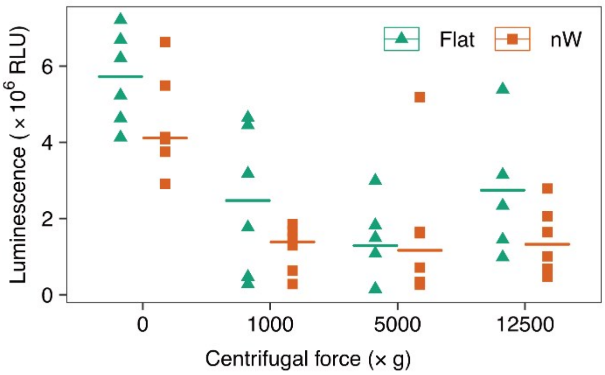

Fig S10. Cell viability assay of *S. aureus*. ATP-detecting BacTiter-Glo™ assay shows reduction in cell viability with increasing centrifugal force. Horizontal lines indicate the median value. n = 6.
